# Changes in peripapillary microvasculature in patients with type 2 diabetes patients: effect of systemic hypertension

**DOI:** 10.1038/s41598-023-46374-8

**Published:** 2023-11-09

**Authors:** Jae-Yun Sung, Kook-Hyung Lee, Ji-Ho Jun, Min-Woo Lee

**Affiliations:** 1 Department of Ophthalmology, Chungnam National University Sejong Hospital, Sejong, Republic of Korea; 2 Department of Ophthalmology, Konyang University College of Medicine, #1643 Gwanjeo-dong, Seo-gu, Daejeon, Republic of Korea

**Keywords:** Diseases, Eye diseases, Retinal diseases

## Abstract

To determine the effect of hypertension (HTN) on the peripapillary microvasculature in type 2 diabetes mellitus (T2DM) patients without diabetic retinopathy (DR). The patients were classified into three groups: the control group (group 1), T2DM group (group 2), and both T2DM and HTN group (group 3). Peripapillary vessel density (VD) was compared using analysis of covariance and linear regression analysis was performed to identify the factors affecting the peripapillary VD. A total of 286 eyes were enrolled: 124 in group 1, 111 in group 2, and 51 in group 3. The peripapillary VDs for the full area were 18.3 ± 0.6, 17.8 ± 1.0, and 17.3 ± 1.2 mm^−1^ in group 1, group 2, and group 3, respectively, which were significantly different after adjustment for age and best-corrected visual acuity (*P* < 0.001). In post hoc analyses, group 1 versus group 2 (*P* < 0.001), group 1 versus group 3 (*P* < 0.001), and group 2 versus group 3 (*P* = 0.001) showed significant differences. In linear regression analysis, HTN (B =  − 0.352, *P* = 0.043) and peripapillary retinal nerve fiber layer (pRNFL) thickness (B = 0.045, *P* < 0.001) were significantly associated with peripapillary VD in T2DM patients. Peripapillary VD in T2DM patients without clinical DR were lower compared to normal controls, and they were more decreased when HTN was comorbid. The combination of ischemic damage by high blood pressure and impairment of the neurovascular unit by hyperglycemia would result in more severe deterioration of peripapillary microvasculature, and this impairment could be also reflected by pRNFL thinning.

## Introduction

Type 2 diabetes (T2DM) can lead to diabetic retinopathy (DR), a major cause of blindness among working-aged individuals worldwide^1,2^. It may also impair visual function by causing retinal damage before clinical DR is expressed. Diabetic retinal neurodegeneration (DRN) is one of the representative forms of retinal damage occurring before clinical DR, which would result in inner retina thinning^3,4^. Previous studies reported the fast reduction of peripapillary retinal nerve fiber layer (pRNFL) thickness in T2DM patients without clinical DR^5,6^. After the development of optical coherence tomography angiography (OCTA), it has been shown that the impairment of peripapillary microvasculature can occur in T2DM patients in the absence of DR, which may be associated with angiopathy resulting from non-enzymatic glycation of free amino groups of proteins, and DRN via neurovascular coupling^3,5,7–9^.

Hypertension (HTN) is a common systemic disease that causes vascular complications, including cardiovascular, cerebrovascular, and renal diseases. It can also lead to severe visual impairment due to hypertensive retinopathy through a vascular remodeling process in retinal arterioles which increases the wall-to-lumen ratio by thickening the arterial wall under uncontrolled blood pressure^10,11^. Even though blood pressure is well controlled, various forms of retinal damage may progress in patients with chronic HTN. Previous studies reported thinner pRNFL in HTN patients with well-controlled blood pressure compared to normal controls^12,13^. Shin et al.^14^ also found impaired peripapillary microvasculature using OCTA in patients with HTN for ≥ 10 years. As such, both T2DM and HTN can damage the peripapillary area of the retina and the damage may be more severe when the two diseases co-exist. However, few studies have reported the impairment of peripapillary microvasculature in patients with concurrent T2DM and HTN in detail.

The purpose of this study was to identify the effect of HTN on the peripapillary microvasculature of T2DM patients via evaluation of the peripapillary vessel density (VD) and perfusion density (PD) using OCTA.

## Methods

### Patients

This retrospective, cross-sectional study received approval from the Institutional Review Board/Ethics Committee of Chungnam National University Hospital, Daejeon, Republic of Korea (no. 2022-12-009). The study followed the tenets of the Declaration of Helsinki.

Patients with T2DM who visited Chungnam National University Hospital for DR evaluation between March 2018 and September 2022 were enrolled, which was a total of 286 patients. T2DM and HTN were diagnosed according to the criteria of the American Diabetes Association (fasting plasma glucose level ≥ 126 mg/dL or 2 h plasma glucose level ≥ 200 mg/dL during a 75 g oral glucose tolerance test, or HbA1c of ≥ 6.5%) and the Korean HTN treatment guideline (clinic blood pressure ≥ 140/90 mmHg; home blood pressure ≥ 135/85 mmHg)^15,16^. Blood pressure in all HTN patients was well controlled. The control group included participants without ophthalmic or systemic diseases. The requirement for obtaining informed consent was waived due to the retrospective nature of the study under confirmation by the Institutional Review Board/Ethics Committee of Chungnam National University Hospital. Complete ophthalmologic examination including best-corrected visual acuity (BCVA), intraocular pressure (IOP), spherical equivalent, and axial length was performed. Subjects were classified into three groups: the control group (group 1), the T2DM group (group 2), and both the T2DM and HTN group (group 3). The exclusion criteria were patients with a history of systemic disease other than T2DM and HTN, ocular surgery other than cataract extraction, ophthalmic disease, IOP > 21 mmHg, axial length ≥ 26.0 mm, and optic disc pathology. Patients with evidence of DR or changes associated with hypertensive retinopathy, including microaneurysm, cotton wool spots, retinal hemorrhage, or disc edema, were also excluded. One eye that met the inclusion criteria was included in the study, and if both eyes of one patient met the inclusion criteria, the eye with the better image quality was selected for analysis. A part of this patient cohort was also included in our previous studies^7,17–19^.

### OCT and OCTA imaging

OCT measurements were obtained using a Cirrus HD OCT 5000 (version 10.0; Carl Zeiss Meditec, Dublin, CA, USA). The pRNFL thickness was measured using a 200 × 200 optic disc cube scanning protocol. An axial scan with a resolution of 200 × 200 pixels was performed on a 6 × 6 mm area centered on the optic nerve head.

**Figure 1:**
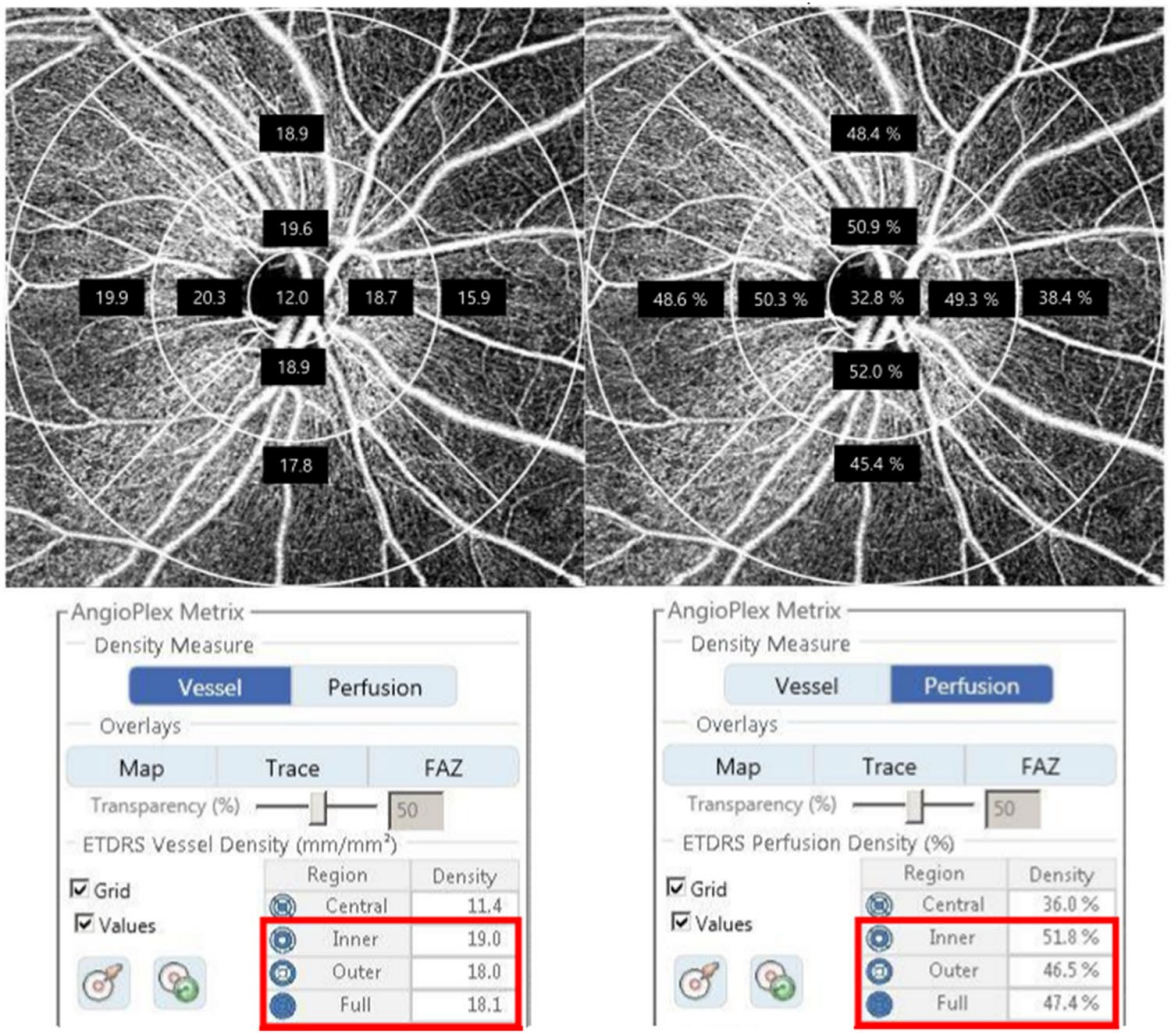
Peripapillary microvasculature in a 6 × 6 mm optical coherence tomography angiography image. Overlay of Early Treatment of Diabetic Retinopathy Study grid on the en-face image of the superficial capillary plexus. The red boxes show automated quantitative measurements of the mean vessel density and perfusion density in the inner ring, outer ring, and full area.

OCTA examination was performed using AngioPlex OCTA platform (Carl Zeiss Meditec), with a wavelength of 840 nm taking 68,000 A-scans/s. The device provides sensitive and accurate information by incorporating the optical microangiography (OMAG) algorithm and retinal tracking technology. Images were obtained using a 6 × 6 mm scan centered on the optic head. The scans were analyzed using en-face OCTA images obtained by the OMAG algorithm in the AngioPlex software. The VD (total length of perfused vasculature per unit area in a region of measurement) and PD (total area of perfused vasculature per unit area of measurement) of the superficial capillary plexus were automatically measured by the software. The software quantified VD and PD as subfields according to the Early Treatment of Diabetic Retinopathy Study (Fig. 1). We analyzed the peripapillary VD and PD in the superficial capillary plexus of the inner and outer rings, and the 6-mm full area described in a previous study^7^. Scans with a signal strength < 9, fixation loss, motion artifacts, or segmentation errors were excluded.

### Statistical analysis

The demographics and OCT and OCTA measurements were compared using one-way analysis of variance with the Bonferroni post hoc test. The chi-square test was used to compare categorical data. OCTA measurements were compared using analysis of covariance to control for the effect of covariate values., including age and BCVA. Univariate and multivariate linear regression analyses were performed to identify factors affecting the peripapillary VD in T2DM patients; we analyzed the VD of the outer ring to exclude the optic disc area. Logistic regression analyses with adjustment for age were performed to determine the OCT and OCTA parameters associated with HTN in T2DM patients. Data analyses were performed using SPSS statistical software (version 18.0; IBM Corp., Armonk, NY, USA).

## Results

### Demographics

A total of 286 eyes were enrolled: 124 in group 1, 111 in group 2, and 51 in group 3. The mean age was 60.2 ± 8.4, 59.0 ± 9.7, and 64.4 ± 7.9 years in group 1, group 2, and group 3, respectively (*P* = 0.002) (Table 1). The BCVA of each group was − 0.02 ± 0.06, + 0.00 ± 0.06, and + 0.00 ± 0.05, respectively (*P* = 0.035). Other characteristics, including sex, spherical equivalent, IOP, and axial length, were not significantly different among the groups.

**Table 1:** Demographics and clinical characteristics in each group.

	Group 1 (n = 124)	Group 2 (n = 111)	Group 3 (n = 51)	*P* value
Age (years)	60.2 ± 8.4	59.0 ± 9.7	64.4 ± 7.9	**0.002**
Sex (men, %)	49 (39.5)	49 (44.1)	28 (54.9)	0.176
T2DM duration (years)	n/a	7.8 ± 6.0	8.2 ± 7.9	0.723
HbA1c (%)	n/a	6.9 ± 0.9	7.1 ± 0.9	0.179
HTN duration (years)	n/a	n/a	9.5 ± 6.1	n/a
Laterality (right, %)	66 (53.2)	57 (51.4)	25 (49.0)	0.875
BCVA (logMAR)	− 0.02 ± 0.06	+ 0.00 ± 0.06	+ 0.00 ± 0.05	**0.035**
Spherical equivalent (diopter)	− 0.27 ± 1.87	− 0.28 ± 1.73	− 0.06 ± 1.56	0.730
Intraocular pressure (mmHg)	14.9 ± 2.8	15.6 ± 3.0	15.8 ± 2.7	0.103
Axial length (mm)	23.6 ± 0.9	23.7 ± 0.8	23.9 ± 1.0	0.127
Central macular thickness (μm)	252.5 ± 18.5	247.4 ± 17.6	25,373 ± 17.6	0.068

Data are mean ± SD unless otherwise indicated.

Values in boldface (*P* < 0.050) are statistically significant.

*T2DM* Type 2 diabetes mellitus, *HTN* Hypertension, *BCVA* Best-corrected visual acuity, *pRNFL* Peripapillary retinal nerve fiber layer.

### pRNFL thickness in each group

The mean pRNFL thicknesses were 97.2 ± 7.1, 95.0 ± 8.1, and 91.4 ± 9.8 μm in group 1, group 2, and group 3, respectively (*P* < 0.001) (Table 2). In post hoc analyses, group 1 versus group 3 (*P* < 0.001) and group 2 versus group 3 (*P* = 0.024) showed significant differences. In sectoral thicknesses, pRNFL thicknesses of superior (*P* = 0.011), temporal (*P* = 0.012), and inferior (*P* = 0.001) sectors were significantly different among the groups.

**Table 2:** Peripapillary retinal nerve fiber layer thickness in each group.

	Group 1	Group 2	Group 3	*P* value
Mean	97.2 ± 7.1	95.0 ± 8.1	91.4 ± 9.8	** < 0.001**
Sector				
Superior	121.3 ± 16.6	118.3 ± 17.9	112.2 ± 21.1	**0.011**
Temporal	73.2 ± 10.3	70.6 ± 11.9	67.4 ± 15.0	**0.012**
Inferior	125.3 ± 14.3	124.5 ± 18.6	114.5 ± 20.1	**0.001**
Nasal	69.1 ± 8.5	68.7 ± 13.0	71.8 ± 15.4	0.276

Data are mean ± SD (μm).

Values in boldface (*P* < 0.050) are statistically significant.

### OCTA parameters in each group

The peripapillary VDs for the full area were 18.3 ± 0.6, 17.8 ± 1.0, and 17.3 ± 1.2 mm^−1^, and VDs of the outer ring were 18.9 ± 0.6, 18.6 ± 1.1, and 17.9 ± 1.5 in group 1, group 2, and group 3, respectively, which was significantly different after adjustment for age and BCVA (both *P* < 0.001) (Table 3). In post hoc analyses, group 1 versus group 2 (*P* < 0.001), group 1 versus group 3 (*P* < 0.001), and group 2 versus group 3 (*P* = 0.001) showed significant differences. The VDs of the outer ring and inner ring in each group were similar to those for the the full area, except for group 2 versus group 3 (*P* = 0.837) of the inner ring.

**Table 3:** Optical coherence tomography angiography parameters in each group.

	Group 1	Group 2	Group 3	*P* value*
VD (mm^−1^)				
Full	18.3 ± 0.6	17.8 ± 1.0	17.3 ± 1.2	** < 0.001**
Outer	18.9 ± 0.6	18.6 ± 1.1	17.9 ± 1.5	** < 0.001**
Inner	17.7 ± 1.2	17.2 ± 1.6	17.1 ± 1.6	**0.007**
PD (%)				
Full	46.7 ± 1.3	45.6 ± 2.9	44.3 ± 3.4	** < 0.001**
Outer	48.0 ± 1.3	47.0 ± 3.3	45.5 ± 4.3	** < 0.001**
Inner	46.4 ± 3.1	45.4 ± 4.2	44.8 ± 4.4	**0.026**

Data are mean ± SD.

Values in boldface (*P* < 0.050) are statistically significant.

*VD* Vessel density, *PD* Perfusion density.

*P value after adjustment for are and best-corrected visual acuity.

**Figure 2:**
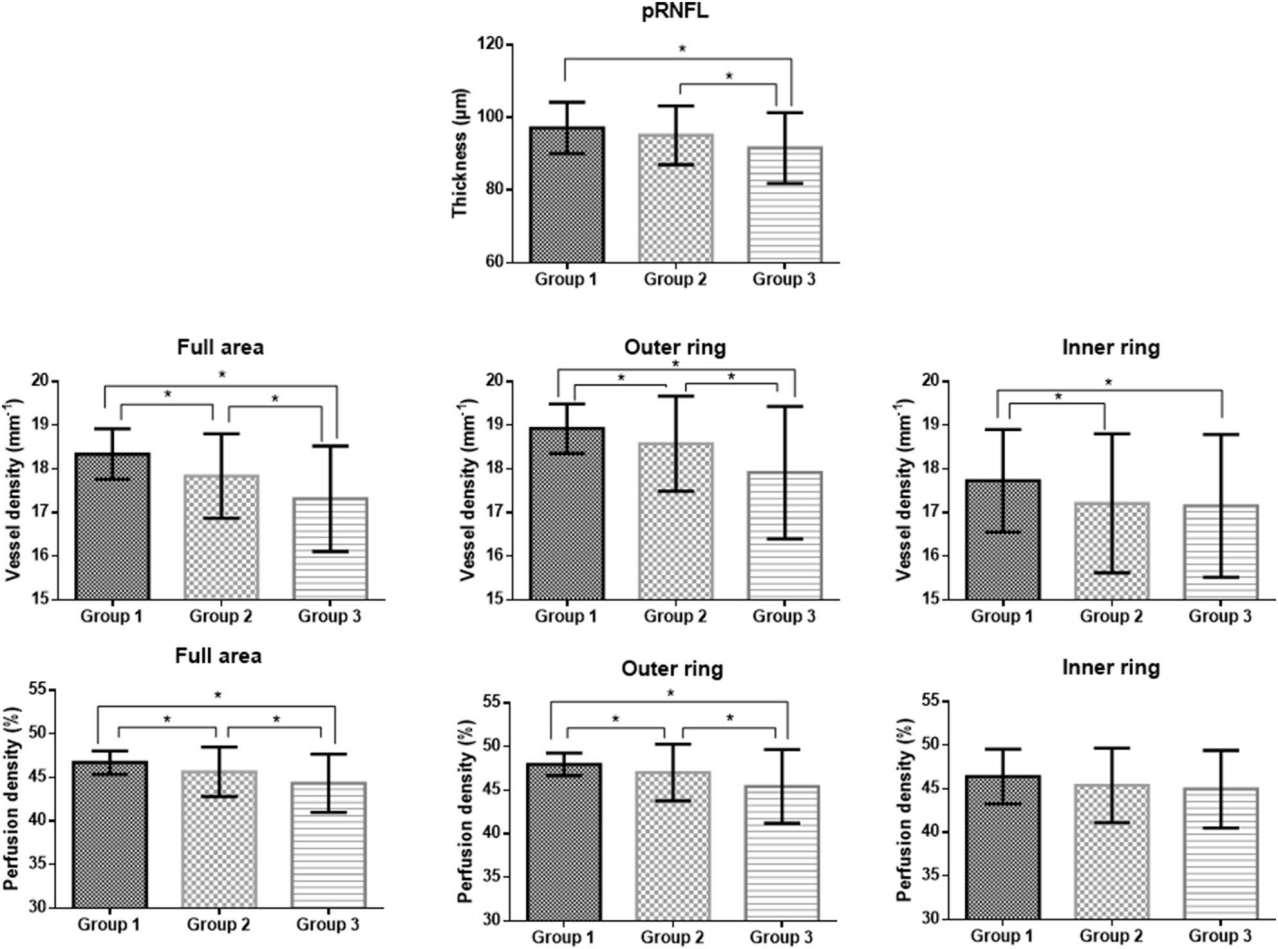
The mean peripapillary retinal nerve fiber layer (pRNFL) thickness, vessel density, and perfusion density in each group. Error bars indicate standard deviation. *Statistically significant results.

The peripapillary PDs of the full area were 46.7 ± 1.3, 45.6 ± 2.9, and 44.3 ± 3.4%, and PD of the outer ring was 48.0 ± 1.3, 47.0 ± 3.2, and 45.4 ± 4.2% in group 1, group 2, and group 3, respectively (both *P* < 0.001). In post hoc analyses, all comparisons were significantly different, except for group 1 versus group 2 (*P* = 0.134) and group 2 versus group 3 (*P* = 0.599) of the inner ring (Fig. 2).

### Linear regression analyses to identify the factors associated with VD in patients with T2DM

In univariate analysis, age (B =  − 0.036, *P* = 0.001), HTN (B =  − 0.647, *P* = 0.003), and pRNFL (B = 0.053, *P* < 0.001) were significant factors associated with peripapillary VD of the outer ring (Table 4). These factors also showed a significant result in multivariate analysis (age, B =  − 0.025, *P* = 0.016; HTN, B =  − 0.352, *P* = 0.043; pRNFL, B = 0.045, *P* < 0.001).

**Table 4:** Univariate and multivariate linear regression analyses for determining the factors associated with peripapillary vessel density in patients with type 2 diabetes.

	Univariate		Multivariate	
	B (95% CI)	*P* value	B (95% CI)	*P* value
Age	− 0.036 (− 0.056, − 0.015)	**0.001**	− 0.025 (− 0.045, − 0.005)	**0.016**
Sex	0.465 (− 0.062, 0.923)	0.092		
T2DM duration	− 0.018 (− 0.048, 0.013)	0.253		
HTN	− 0.647 (− 1.069, − 0.226)	**0.003**	− 0.352 (− 0.764, − 0.010)	**0.043**
HbA1c	− 0.196 (− 0.421, 0.029)	0.088		
BCVA	− 1.112 (− 4.684, 2.448)	0.537		
SE	− 0.112 (− 0.231, 0.007)	0.065		
IOP	− 0.030 (− 0.099, 0.040)	0.400		
Axial length	− 0.243 (− 0.451, 0.004)	0.060		
CMT	− 0.003 (− 0.014, 0.009)	0.641		
pRNFL	0.053 (0.031, 0.074)	** < 0.001**	0.045 (0.024, 0.066)	** < 0.001**

Values in boldface (*P* < 0.050) are statistically significant.

*T2DM* Type 2 diabetes mellitus, *HTN* Hypertension, *BCVA* Best-corrected visual acuity, *SE* Spherical equivalent, *IOP* Intraocular pressure, *CMT* Central macular thickness, *pRNFL* Peripapillary retinal nerve fiber layer.

### Logistic regression analyses to determine the OCT and OCTA parameters associated with HTN in patients with T2DM

In logistic regression analyses with adjustment for age, pRNFL (OR = 0.958, *P* = 0.040) and peripapillary VD of the outer ring (OR = 0.751, *P* = 0.044) were significant factors associated with HTN in patients with T2DM (Table 5).

**Table 5:** Logistic regression analyses after adjustment for age to determine the OCT and OCTA parameters associated with hypertension in patients with type 2 diabetes.

	B	OR (95% CI)	*P* value
CMT	0.023	1.023 (0.991, 1.044)	0.060
pRNFL	− 0.043	0.958 (0.920, 0.998)	**0.040**
VD			
Full	− 0.312	0.732 (0.527, 1.017)	0.063
Outer	− 0.286	0.751 (0.569, 0.993)	**0.044**
Inner	− 0.001	0.999 (0.804, 1.242)	0.994
PD			
Full	− 0.095	0.910 (0.814, 1.016)	0.095
Outer	− 0.077	0.926 (0.844, 1.015)	0.101
Inner	− 0.017	0.983 (0.907, 1.065)	0.672

Values in boldface (*P* < 0.050) are statistically significant.

*CMT* Central macular thickness, *pRNFL* Peripapillary retinal nerve fiber layer, *VD* Vessel density, *PD* Perfusion density.

## Discussion

As the morphology and physiology of retinal microvasculature share similar features with cardiac and cerebral vasculature, the retina may represent an accessible window to detect microvascular change occurring in the setting of systemic diseases and neurodegenerative disorders^20–23^. Thus, evaluation of the retinal microvasculature is important in such patients, which can be assessed using OCTA. In this study, we aimed to identify the effect of HTN on peripapillary microvasculature in T2DM patients using OCTA and found out that peripapillary VD and PD of T2DM patients with HTN were significantly lower than those of T2DM patients without HTN. Additionally, HTN and pRNFL thickness were significant factors associated with peripapillary VD in T2DM patients.

Recent studies indicated that T2DM patients without DR have lower peripapillary perfusion than normal controls. Vujosevic et al.^5^ found a significant decrease in VD, number of branches, and total branch length in the peripapillary area in patients with DM, even without clinical signs of DR when compared with healthy subjects. Shin et al.^8^ reported that peripapillary microvascular parameters in the no DR and NPDR groups, including VD and PD, were lower than those of normal controls using OCTA. Our study showed that the peripapillary VD and PD were significantly lower in group 2 and group 3 than in group 1, which was consistent with previous studies. Early damage to the peripapillary microvasculature before DR changes may be associated with its peculiar anatomy, characterized by capillaries with long straight paths and rare anastomotic connections^5,24^. Such impairment of peripapillary microvasculature in T2DM patients without DR can suggest an early preclinical sign of diabetic microvascular complications. Yuan et al.^25^ found that lower peripapillary VD and vessel length density of superficial capillary plexus were significantly associated with increased risk for DR incidence among the T2DM population. Therefore, evaluation of the peripapillary microvasculature can be an important factor in predicting progression to DR and assessing the degree of retinal damage in T2DM patients without DR.

Although decreased peripapillary perfusion in T2DM patients without DR has been widely studied, there is limited evidence for the change of peripapillary perfusion in patients with HTN without hypertensive retinopathy. Peng et al.^26^ found that the VD of radial peripapillary capillaries was not significantly different between HTN patients without hypertensive retinopathy and normal controls. Hua et al.^27^ also reported no significant difference in the peripapillary capillary density between HTN patients with intensive blood pressure control and normal controls. In the study of Shin et al.^14^, changes in peripapillary VD and PD were correlated with the duration of HTN. They found no significant differences in peripapillary VD and PD in patients with HTN for < 10 years compared to normal controls, but lower peripapillary VD and PD in patients with HTN for ≥ 10 years. On the other hand, our study showed that T2DM patients with HTN, having a history of mean HTN duration for < 10 years without hypertensive retinopathy, had significantly lower peripapillary VD and PD than patients with T2DM only and normal controls. Additionally, HTN was a significant factor affecting peripapillary VD in T2DM patients, and peripapillary VD of the outer ring was significantly associated with HTN in T2DM patients. Therefore, the combination of HTN and T2DM would result in more severe deterioration of peripapillary microvasculature.

HTN can cause microvascular damage such as vascular remodeling which can cause increased resistance and rigidity. Additionally, sustained vasospasm of the retinal arterioles reflecting vasoconstriction as an autoregulatory response to HTN would cause compression of the venules and lead to decreased peripapillary microvasculature^28^. This impaired retinal-vascular autoregulation in response to high blood pressure can be more severe in patients with T2DM, leading to a lower capacity to regulate retinal blood flow and decreased peripapillary microvasculature^29^. Additionally, HTN promotes retinal capillary endothelial damage and causes increased expression of vascular endothelial growth factor, which is the main pathophysiological insult leading to the progressive changes of DR^30,31^. Hyperglycemia also causes endothelial damage, which alters endothelial cell matrix production enhancing endothelial collagen IV and fibronectin production and increasing the activity of enzymes involved in collagen synthesis, and they could result in generalized endothelial thickening^30,32,33^. In addition, elevated levels of circulating advanced glycation end products in the presence of hyperglycemia contribute to vascular remodeling processes associated with inflammation and apoptosis^9^. As such, both diseases target the endothelium, which may cause weakening of repair processes in vascular remodeling. These mechanisms may be associated with severe impairment of peripapillary microvasculature in T2DM patients with HTN, and also support explaining the fact that HTN is an independent risk factor for both the initial development of DR and its progression^34–36^.

The pRNFL was significantly thinner in T2DM patients with HTN than in T2DM patients and controls, which was consistent with the results of a previous study^37^. Additionally, similar to the peripapillary VD of the outer ring, pRNFL thickness was a significant factor associated with HTN in patients with T2DM. Previous studies reported a significant correlation between pRNFL thickness and peripapillary microvasculature in T2DM patients, and the reduction of pRNFL thickness would be related to the impairment of peripapillary microvasculature^5,7^. The inner retina and retinal microvasculature are linked as neurovascular coupling, and the radial peripapillary plexus is known as the most important structure in maintaining RNFL integrity^38^. Decreased peripapillary perfusion due to impaired autoregulation associated with DRN and HTN can cause ischemic damage to the inner retina, and such damage to the inner retina may induce the deterioration of neurovascular coupling, leading to the impairment of peripapillary microvasculature. As such, pRNFL and peripapillary microvasculature would be influenced by each other constantly and also reflect each other’s condition. Further studies are needed to identify the sequential relationship between the two processes and which one has a stronger influence.

This study had several limitations. First, the retrospective design may have introduced some selection bias. Second, we could not completely rule out the possibility that group 3 enrolled HTN patients with a previous occurrence and subsequent regression of hypertensive retinopathy which could have affected the peripapillary microvasculature. Third, we did not perform various visual function tests that could be related to peripapillary microvasculature impairment. Nevertheless, to the best of our knowledge, this is the first study to investigate the impact of HTN on peripapillary microvasculature in T2DM patients without clinical DR in detail. Another strength of this study is that we included OCTA images with signal strength ≥ 9 to allow accurate analyses.

In conclusion, peripapillary VD and PD were lower in T2DM patients without clinical DR compared to normal controls, and the values were particularly lower when the patients had comorbid HTN. HTN significantly affected the peripapillary VD in T2DM patients, and the pRNFL thickness and peripapillary VD of the outer ring were associated with HTN in T2DM patients. The combination of ischemic damage by high blood pressure and impaired neurovascular unit by hyperglycemia may result in more severe deterioration of peripapillary microvasculature; this impairment could be reflected as pRNFL thinning, which is linked to the peripapillary microvasculature through neurovascular coupling. These findings suggest that HTN is a risk factor for DR progression in T2DM patients and emphasize the importance of monitoring peripapillary microvasculature in these patients.

## Data Availability

The datasets used and/or analyzed during the current study available from the corresponding author on reasonable request.
